# Familial Accumulation of Social Anxiety Symptoms and Maladaptive Emotion Regulation

**DOI:** 10.1371/journal.pone.0153153

**Published:** 2016-04-07

**Authors:** Julia Asbrand, Jennifer Svaldi, Martina Krämer, Christoph Breuninger, Brunna Tuschen-Caffier

**Affiliations:** 1 Department of Clinical Psychology and Psychotherapy, Institute of Psychology, University of Freiburg, Freiburg, Germany; 2 Department of Clinical Psychology, Institute of Psychology, University of Tübingen, Tübingen, Germany; Technion - Israel Institute of Technology, ISRAEL

## Abstract

**Background:**

Social anxiety is thought to be strongly related to maladaptive emotion regulation (ER). As social anxiety symptoms accumulate in families, we hypothesize that maladaptive ER is also more prevalent in families with anxious children. Thus, we analyze differences in emotion regulation of both child and mother in relation to social anxiety, as well as both their ER strategies in dealing with anxiety. Further, a positive relation between child and maternal ER strategies is assumed.

**Method:**

Children (aged 9 to 13 years) with social, anxiety disorder (SAD; *n* = 25) and healthy controls (HC, *n* = 26) as well as their mothers completed several measures of social anxiety and trait ER strategies towards anxiety. As ER of children is still in development, age is considered as covariate.

**Results:**

SAD children and their mothers reported more maladaptive ER strategies than HC dyads. Maternal maladaptive ER was related negatively to child adaptive ER which was further moderated by the child’s age.

**Discussion:**

Maladaptive ER strategies seem to contribute to the exacerbation of social anxiety in both mother and child. Mothers reporting maladaptive ER may have difficulties supporting their child in coping with social anxiety while simultaneously also experiencing heightened levels of anxiety. Deeper understanding of interactional processes between mothers and children during development can assist the comprehension of factors maintaining SAD. Implications for future research and possible consequences for interventions are discussed.

## Introduction

Social anxiety disorder (SAD) is one of the most frequent mental disorders in childhood (e.g. [1]). The onset of the disorder typically occurs in late childhood or early adolescence [1–3]. Maintaining factors in SAD, at least in adults, have been shown to be largely cognitive in nature (e.g., [4, 5]). In addition to cognitive factors, deficits in emotion regulation (ER) have been associated with SAD in adults (e.g. [6, 7]). ER includes internal and external processes aimed to maintain or modulate the occurrence of emotion as (e.g. [8]), for example by increasing or decreasing the experienced intensity of an emotion. Due to its early onset, a stronger focus on childhood SAD and deficits in ER is needed to include developmental peculiarities.

Children with anxiety disorders in general report a heightened intensity of negative emotion [9] as well as impairments in ER [10]. For example, a study by Southam-Gerow and Kendall [11] found youths with SAD to be less aware of possibilities to change emotionally challenging situations than non-anxious youths. From a developmental perspective, ER begins as an interactive process between parent and child [12, 13]. Parents support the infant’s self-regulation of emotions, e.g. by soothing it when upset. Thereby, they substantially participate in the child's development of ER strategies. The underlying mechanisms can be manifold. For example, parents influence children directly by–among others–modeling ER strategies, reacting to the child's emotions, and social referencing (e.g. [12]). Further, parental influence can be indirect, e.g. via family climate such as marital relation (e.g. [14, 15]). In a tripartite model of familial influence, Morris et al. [15] lay out a model for the development of ER in children influenced by general familial influences such as modeling and observational learning, parenting practices concerning emotion and emotion management, and the emotional climate of the family. Importantly, the authors include parental characteristics such as mental health as well as child characteristics as further influential factors.

Symptoms of clinically relevant social anxiety are often found in both parent and child (e.g. [16–18]), implying familial accumulation of social anxiety symptoms. The same is true for ER, with previous studies showing a positive link between parental ER modeling of reappraisal and child reappraisal in preschool children [19] as well as a positive relation between parental and child expressive suppression in youths [20]. However, only few studies on familial accumulation of maladaptive ER (e.g. [21, 22]) or anxiety (e.g. [16–18]) have been conducted. For instance, in a community sample of children between ages 7 and 12, both mothers and children reported on psychopathological symptoms [23]. For internalizing symptoms, the association between child ER and child internalizing symptoms was strongest when parental reactions were mostly supportive. Furthermore, child ER mediated the association between maternal psychopathology and child psychopathology. That is, if mothers model a restricted range of emotions, mostly negative in nature, their children show a similar pattern [23, 24]. Whether maternal ER has a similar mediating role remains an open question. Additionally, models of the links between maternal and child psychopathology might differ between specific disorders as well as between different stages of development. Bariola, Hughes and Gullone [20] did not find age to moderate the association of parental and child ER. However, the assessed age span was relatively large (10–20 years) and, thus, might have focused too strongly on youths instead of children. Little is known about ER and anxiety in late childhood (9–13 years). Considering SAD, the age between 9 and 13 is especially interesting as the disorders’ earliest onset is reported in late childhood [2]. Based on the tripartite model of familial influence [15], age should be controlled for as a possible factor in intergenerational transmission.

In line with the extant research, the aim of the present study was to assess ER strategies in children with SAD and their parents and their relation to social anxiety symptoms. Previous research has linked some strategies (e.g., expressive suppression) to psychopathology, and others to psychological well-being (e.g., cognitive reappraisal; for an overview see [25]). Further studies have emphasized the importance of flexible management of the various ER strategies [26]. Therefore, the present study considered several strategies which were overall summarized as adaptive vs. maladaptive. Based on theoretical models and previous research, it was first hypothesized that children with SAD as well as their mothers would report more maladaptive ER strategies than healthy control children and their mothers (*Emotion Regulation*). Further, we expected a positive relation between maternal and child maladaptive ER (*Familial relations in Emotion Regulation*).

## Method

### Participants

Parents of children (aged 9 to 13) were recruited by advertisements in local newspapers, medical facilities and information handed out in schools as part of a larger project sponsored by the German Research Foundation (DFG; TU 78/5-2). SAD symptoms and symptoms of further psychopathology were first screened by means of a telephone interview with interested families (*n* = 188). If the screening assessment suggested eligibility, families (*n* = 99) were invited to a diagnostic interview (Kinder-DIPS; [27]; a modified and extended version of the Anxiety Disorders Interview Schedule for children, ADIS-C; [28]) which assessed common mental disorders in children and youth. Reliability and validity of the interview were confirmed in German samples and classified as good [27]. For a diagnosis on DSM-IV criteria, child and parent reports were combined with clinical impressions and questionnaire data by both interviewers under supervision by a trained therapist. After the diagnostic interview, 34 children fulfilled the criteria for a SAD diagnosis, while 28 children did not report any lifetime mental disorder (healthy controls, HC) and were thus included in the study. Of these included families, 55 agreed to participate in the current study (SAD: *n* = 28, HC: *n* = 27). Due to technical problems, the data from 4 children had to be excluded, leaving a sample of 51 mother-child dyads (25 SAD, 26 HC).

For participation, children were reimbursed with vouchers worth 10€, while parents were given 20€. Following completion of the study, SAD children were offered participation in a group treatment of SAD. After receiving written and oral information about the project, both children and parents provided their written consent. The study was approved by the local ethics committee (Ethik-Kommission Freiburg; 517/14).

### Procedure

In a diagnostic session with the child and the mother, all dyads were administered a structured clinical interview to assess existence of the clinical diagnosis of SAD and non-existence of lifetime mental illness, respectively. After the diagnostic session, both mother and child filled in questionnaires on sociodemographic variables and psychopathology. Questionnaires on ER were administered during a testing session which took place in families' homes. Before filling in the ER questionnaires, children underwent light exercise and changes of position as a physiological challenge task as well as a puzzle task; the procedure and results are presented elsewhere (Asbrand, Blechert, Nitschke, Tuschen-Caffier, & Schmitz, submitted).

### Materials

#### Child

The Social Anxiety Scale for Children–Revised (SASC-R [29]) measures social anxiety symptoms by child self-report (22 items, e.g. “I get nervous when I talk to new kids”) with total scores ranging from 18 to 90. An adapted version for parental assessment was additionally used [30]. Both children and parents respond to each item using a 5-point Likert-type scale ranging from 1 (not at all) to 5 (all the time). The SASC-R has satisfactory test-retest reliability (0.67) and internal consistency (0.76; [31]). Moderate correlation has been confirmed with general measures of anxiety, self-perceptions of social confidence, teacher ratings of anxiety withdrawal, and peer ratings of popularity [32]. The internal consistency of the SASC-R in the current sample was excellent (child: α = .96, mother: α = .98).

#### Mother

The Symptom Checklist Short (SCL-K-9, [33] a short version of the SCL-90-R [34], includes 9 items to economically screen for the most common psychopathological symptoms in adults (e.g. anxiety, depression etc.). Symptoms experienced in the past week (e.g., “How often did you feel like you were worrying too much?”) are assessed on a 5-point Likert-like scale (“not at all” to “very often”). Internal consistency for the questionnaire is excellent (Cronbach’s *α* = .87). Convergent validity has been established by correlation with similar questionnaires [33]. Internal consistency for the SCL-K-9 in the current sample was good (Cronbach’s *α* = .79).

The Mini-Social Phobia Inventory (Mini-SPIN, [35]) serves as screening instrument for generalized social anxiety, using three items (e.g., “Being embarrassed or looking stupid are among my worst fears”) which are answered on a 6-point scale (0 “not at all” to 5 “extremely”). A cut-off of 6 is suggested to separate moderate from low symptoms of social anxiety [35]. Sensitivity (94.6%) and specificity (90.4%) at this cut-off are good [35]. Internal consistency in the current sample was excellent (Cronbach’s *α* = .91).

#### Child and Mother

The Fragebogen zur Erhebung der Emotionsregulation bei Kindern und Jugendlichen (FEEL-KJ; Questionnaire on Emotion Regulation in Children and Youth, [36])is a German trait questionnaire covering a broad range of ER strategies over 30 items rated on a 5-point scale concerning frequency of strategy application (1 "almost never" to 5 "almost always"). The same items are used to assess coping with anxiety, anger and sadness. In the current study, only the questionnaire covering anxiety was used. From the items, 15 strategies are extracted which can be classified as adaptive emotion regulation strategies (ER-S; e.g., “If I am anxious, I try to remember happy times”; problem-oriented action, cheering up, distraction, acceptance, cognitive problem solving, forgetting, reappraisal) and maladaptive ER-S (e.g., “If I am anxious, I start a fight with someone else”; withdrawal, self-degradation, resigning, perseveration, aggression). Three further strategies (suppression, social support, emotion expression) did not load on either factor in the original study [36]. While suppression is often interpreted as maladaptive (e.g. [37]), it did not load on the same factor as other maladaptive strategies in the validation study [36]. Internal consistencies for the strategies were satisfactory (α = .69) to excellent (α = .91). Re-test reliability was also confirmed to be good (after six weeks: .62 ≤ *rtt* ≤ .81; [36]). Internal consistency in the current sample was excellent for child adaptive ER-S (α = .85) and maladaptive ER-S (α = .81).

To achieve comparability of ER-S between mother and child, we constructed a maternal trait version of the FEEL-KJ. This consisted of the same items as the FEEL-KJ but used an adapted introduction. Comparisons with an established questionnaire for assessment of ER-S in adults (Emotion Regulation Questionnaire, ERQ; [38]) showed significant correlations between adaptive ER-S and reappraisal (*r* = .488, *p* < .001) as well as between maladaptive ER-S and suppression (*r* = .350, *p* = .013) in our sample. Internal consistency in the current sample was again excellent for maternal adaptive ER-S (α = .81) and maladaptive ER-S (α = .75).

### Statistical Analyses

To analyze group differences in ER-S, separate MAN(C)OVAs were calculated for child and mother. Each MAN(C)OVA included Group (SAD, HC) as a factor and both child and maternal adaptive strategies and maladaptive strategies as dependent variables. For child ER-S, age was included as covariate as age has been discussed as an important factor in ER [20, 39].

To examine familial links between child and maternal ER-S, we computed multiple regressions using child maladaptive and adaptive ER-S, respectively, as dependent variables (criterions). As ER-S are still in development during childhood and adolescence, age (in months) was included in the analysis [39] as a continuous variable. Thus, predictors in each multiple regression consisted of z-standardized maternal adaptive and maladaptive ER-S, z-standardized age (in months) and interaction terms to analyze moderator effects. Interaction terms were calculated by multiplying z-standardized maternal maladaptive and adaptive ER-S with z-standardized age variables. All predictors were included in the regression using a full model approach. Multiple regressions were preferred to multiple correlations as regressions can address differential relations between predictor and criterion in different groups, thus examine moderation effects (see [40]). Post-hoc power analyses provide further insight about statistical value of the calculations based on current literature [41].

## Results

### Participants’ characteristics

As shown in Table 1, children in both groups did not differ in terms of age, gender, or type of school. Children in the SAD group and their mothers reported significantly higher child social anxiety than in the HC group. Similarly, mothers of SAD children showed higher social anxiety than mothers of HC children even though the mean did not exceed the cut-off of 6 for clinical social anxiety [35].

**Table 1:** Participant Characteristics.

	Target person	SAD	HC	
		*M (SD)*	*M (SD)*	Statistics
n		25	26	
Age (in years)		10.9 (1.27)	11.1 (1.45)	*t*(49) = 0.62, n.s.
% female		69.2	64.0	*χ*^*2*^(1) = 0.69, n.s.
% elementary school^a^		28.0	19.2	*χ*^*2*^(4) = 3.69, n.s.
SASC-R (child report)	Child	46.4 (12.6)	28.2 (9.65)	*t*(49) = -5.81***
SASC-R (maternal report)	Child	61.3 (13.6)	27.0 (5.11)	*t*(29.0) = -11.7***
Mini-SPIN (maternal report)	Mother	4.26 (3.21)	1.54 (1.63)	*t*(31.7) = -3.81***
SCL-K-9 (mother)	Mother	14.93 (4.10)	12.37 (3.65)	*t*(54) = -2.46*

*Note*. SASC-R = Social anxiety scale for children–revised, Mini-SPIN = Mini–Social Phobia Inventory, SCL-K-9 = Symptom Checklist 9 Items

* p < .05

** p < .01

*** p < .001

^a^ All other children attended secondary school.

### Emotion Regulation

#### Child

A MANCOVA on Group (SAD, HC) with age as a covariate age and child adaptive and maladaptive ER-S as dependent variables revealed a significant main effect of Group, Wilks’ λ = .786, *F*(2,47) = 6.39, *p* = .004, *η*_*p*_^2^ = .214, and Age, Wilks’ λ = .831, *F*(2,47) = 4.78, *p* = .013, *η*_*p*_^2^ = .169. Follow-up univariate ANOVAs showed the group effect to be significant for maladaptive child ER-S, *F*(1,48) = 11.20, *p* = .002, *η*_*p*_^2^ = .189, but not for adaptive child ER-S, *F* < 1.44, *p* > .235. Further, a significant effect of Age was found for adaptive child ER-S, *F*(1,48) = 6.93, *p* = .011, *η*_*p*_^2^ = .126, but not for other strategies, *F*s < 2.85, *p*s > .099. Thus, SAD children self-report more frequent use of maladaptive strategies than HC children (namely, withdrawal, resigning, and perseveration) whereas no differences were found with regard to the usage of adaptive ER-S. Furthermore, in both groups were child adaptive ER-S positively influenced by age pointing to an increasing use of adaptive strategies as children get older.

#### Mother

A MANOVA on Group (SAD, HC) including maternal maladaptive and adaptive ER-S as dependent variables revealed a significant main effect of Group, Wilks’ λ = .749, *F*(2,48) = 8.04, *p* < .001, *η*_*p*_^2^ = .251. Follow-up univariate ANOVAs revealed that the effect was significant for maladaptive maternal ER-S, *F*(1,49) = 16.41, *p* < .001, *η*_*p*_^2^ = .254, but not for adaptive maternal ER-S, *F* < 0.01, *p* > .995 (as reported in Table 2). Hence, mothers of SAD children self-report more maladaptive ER-S than mothers of HC children (namely, withdrawal, resigning and self-degradation; see Table 2). Post-hoc power-analyses for both MAN(C)OVAs showed excellent power, β ≥ .99.

**Table 2:** Group differences in emotion regulation strategies in children and mothers.

	Child					Mother				
	SAD	HC	Statistics^a^			SAD	HC	Statistics		
	*M (SD)*	*M (SD)*	*F/t*	*p*	*η*^*2*^_*p*_*/d*	*M (SD)*	*M (SD)*	*F/t*	*p*	*η*^*2*^_*p*_*/d*
**Adaptive Strategies**	37.7 (9.73)	41.5 (10.73)	1.44	.236	.029	48.3 (7.70)	47.8 (6.50)	0.00	.996	.000
problem-oriented action	5.1 (1.86)	5.8 (2.02)	1.27	1.00	.361	7.3 (1.65)	7.8 (1.52)	1.28	.207	.315
cheering up	5.8 (2.30)	5.7 (2.18)	-0.17	1.00	.045	6.4 (2.08)	6.2 (1.80)	-0.38	.707	.103
distraction	5.8 (2.28)	6.3 (2.41)	0.83	1.00	.213	6.1 (2.18)	6.2 (1.80)	0.07	.948	.050
acceptance	4.8 (2.01)	6.5 (2.04)	2.92	.035	.839	7.0 (1.57)	7.1 (1.70)	0.26	.799	.061
cognitive problem solving	5.9 (2.20)	5.8 (1.80)	-0.20	1.00	.050	8.2 (1.25)	8.2 (1.52)	-0.02	.987	.000
oblivion	5.9 (2.13)	5.8 (1.87)	-0.06	1.00	.050	6.8 (1.09)	6.4 (1.06)	-1.12	.270	.372
reappraisal	4.4 (1.98)	5.6 (2.32)	2.01	.350	.556	6.2 (1.55)	6.0 (1.44)	-0.38	.704	.134
**Maladaptive Strategies**	24.7 (8.20)	18.0 (6.95)	11.20	.002	.189	28.5 (4.87)	22.5 (6.05)	16.41	< .001	.251
withdrawal	4.8 (1.97)	3.1 (1.93)	-3.16	.015	.872	5.3 (1.89)	3.8 (1.56)	-3.21	.002	.866
self-degradation	5.1 (2.19)	4.1 (1.60)	-1.95	.114	.521	7.5 (1.73)	5.5 (1.94)	-3.84	< .001	1.088
resigning	4.9 (2.45)	3.1 (1.80)	-3.01	.016	.837	5.2 (1.52)	4.0 (1.82)	-2.55	.014	.716
perseveration	6.5 (2.65)	4.7 (2.05)	-2.70	.027	.760	6.7 (1.75)	5.8 (1.99)	-1.59	.119	.480
aggression	3.4 (1.96)	3.0 (1.59)	-0.81	.424	.224	4.0 (1.79)	3.4 (1.33)	-1.57	.122	.381

^a^ Post-hoc tests Bonferroni-corrected.

### Familial relations in emotion regulation

#### Child maladaptive ER-S

**Fig 1:**
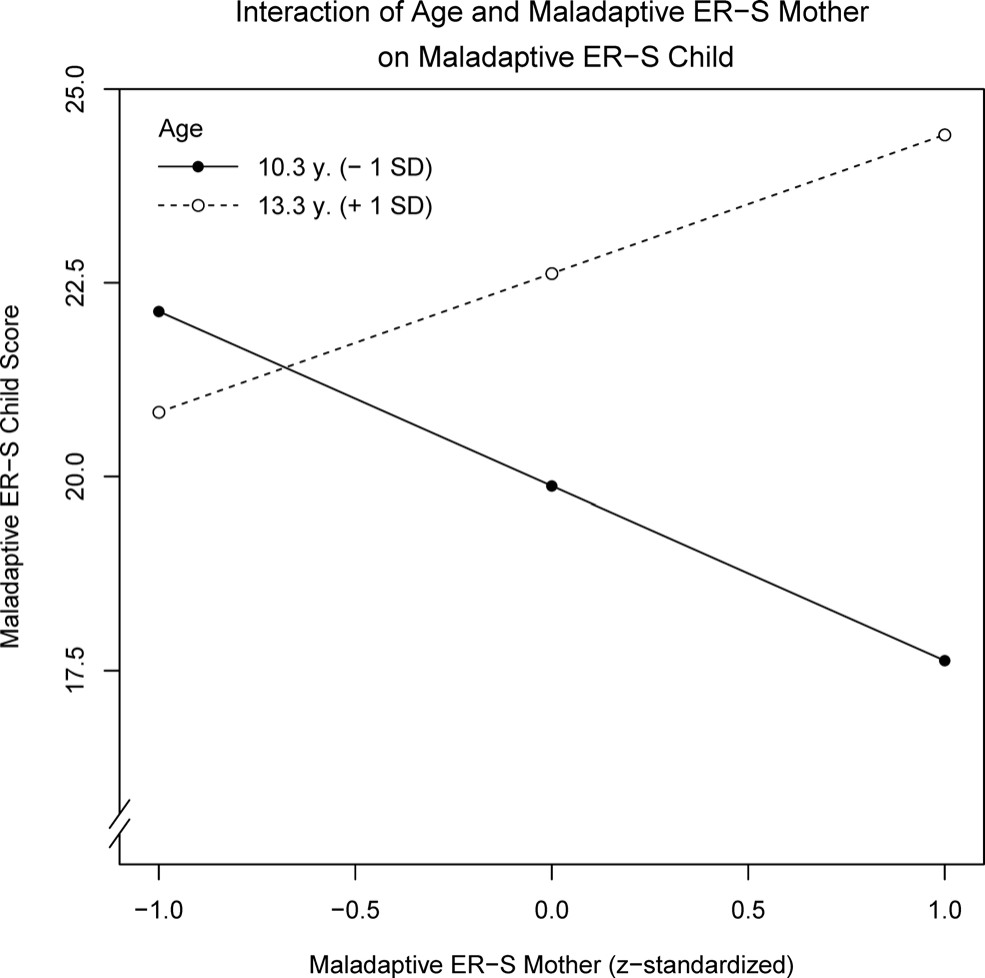
Prediction of child maladaptive ER-S by the interaction of age and maternal maladaptive ER-S. If children are younger, maternal maladaptive ER-S is negatively related to child maladaptive ER-S. In older children, child maladaptive ER-S is positively related to maternal maladaptive ER-S. *Note*. ER-S = Emotion Regulation strategies.

A multiple regression analysis was performed with child maladaptive ER-S as the criterion and predictors consisting of z-standardized maternal adaptive and maladaptive ER-S, z-standardized Age, z-standardized social anxiety and all interaction terms for ER strategies and Age using a full model approach. The overall model explained 17.7% of the variance, *F*(6,42) = 2.72, *p* = .026 (see Table 3). The score of social anxiety symptoms significantly predicted use of maladaptive child ER-S while the interaction term age*maternal maladaptive ER-S showed a trend towards significance (see also Fig 1). A post-hoc power analysis revealed sufficient power, β = .68, in line with empirical tests of power in published studies [41].

**Table 3:** Hierarchical prediction of maladaptive child ER-S by maternal ER-S and age (including first order as well as interaction terms).

		*Model 1*: *Predicting maladaptive child ER-S (R*^*2*^ *=* .177*)				
Block	added Predictors	*ΔR*^*2*^	*b*	*SE*	β	*p*
*1*	SASC-R	.196**	**3.60**	**1.06**	**.443**	**.001**
2	age	.015	1.00	1.05	.124	.349
*3*	maternal maladaptive ER-S	.001	-0.30	1.27	-.036	.816
*4*	maternal adaptive ER-S	.010	-0.86	1.14	-.100	.456
*5*	age*maternal maladaptive ER-S ^a^	.056^†^	1.97	1.08	.244	.075
*6*	age*maternal adaptive ER-S	.001	-0.23	1.06	-.218	.829

*Note*. ER-S = ER strategies, SASC-R = Social Anxiety Scale for Children–Revised

^a^ The term age*maternal maladaptive ER-S predicting child maladaptive ER-S is shown in Fig 1.

#### Child adaptive ER-S

**Fig 2:**
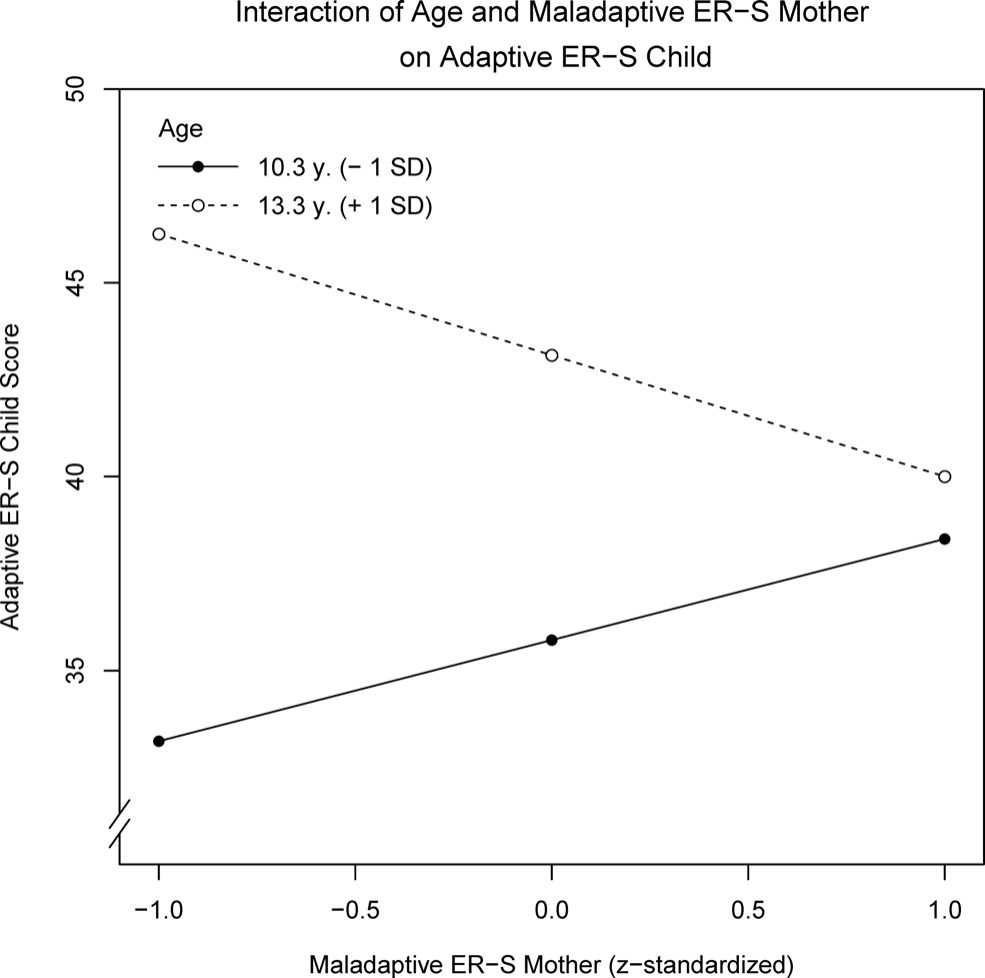
Prediction of child adaptive ER-S by the interaction of age and maternal maladaptive ER-S. If children are younger, maternal maladaptive ER-S is positively related to child adaptive ER-S. In older children, child adaptive ER-S is negatively related to maternal maladaptive ER-S. *Note*. ER-S = Emotion Regulation strategies.

A multiple regression analysis with the same predictors as above, but child adaptive ER-S as the criterion, showed that child adaptive ER-S was significantly predicted by age and by the interaction term age* maternal maladaptive ER-S. No other predictor reached significance. The overall model explained 19.3% of the variance, *F*(6,42) = 2.91, *p* = .018 (see Table 4). Thus, in accordance with the MANCOVA results, child adaptive ER-S use is positively related to age. Additionally, this relation is further negatively influenced by maternal maladaptive ER-S (see also Fig 2). Therefore, age serves as a moderator of the relation between child adaptive ER-S and maternal maladaptive ER-S_._ A post-hoc power analysis revealed sufficient power, β = .62, in line with empirical tests of power in published studies [41].

**Table 4:** Hierarchical prediction of adaptive child ER-S by maternal ER-S and age (including first order as well as interaction terms).

			*Model 2*: *Predicting adaptive child ER-S (R*^*2*^ *=* .193*)			
Block	added Predictors	*ΔR*^*2*^	*b*	*SE*	β	*p*
*1*	SASC-R	.026	-1.66	1.49	-.160	.271
2	age	.158**	**4.08**	**1.37**	**.398**	**.005**
*3*	maternal maladaptive ER-S	.001	-0.36	1.65	-.035	.826
*4*	maternal adaptive ER-S	.035	-2.04	1.45	-.187	.168
*5*	age*maternal maladaptive ER-S ^a^	.074*	**-2.88**	**1.36**	**-.281**	**.040**
*6*	age*maternal adaptive ER-S	.000	-0.04	1.33	-.004	.976

*Note*. ER-S = ER strategies, SASC-R = Social Anxiety Scale for Children–Revised

^†^ p < .1

* p < .05

** p < .01

^a^ The term age*maternal maladaptive ER- predicting child adaptive ER-S is shown in Fig 2.

## Discussion

The current study aimed to examine ER strategies used by mothers and children to deal with anxiety as well as relations of social anxiety and ER. As expected, we found more maladaptive ER strategies in children with SAD and their mothers. Further, use of adaptive strategies in both groups was influenced by age, with older children using more adaptive ER strategies than younger children. Social anxiety symptoms predicted maladaptive ER strategies in children. Children’s adaptive ER strategies, however, were predicted by maternal maladaptive ER strategies moderated by age. If children are younger, more maternal maladaptive ER is related to more child adaptive ER. In older children, conversely, more maternal maladaptive ER is related to less child adaptive ER.

Previous research found increased levels of social anxiety in mothers if their offspring showed heightened social anxiety (e.g. [16–18]). In addition to replicating these findings on anxiety, we found both affected children and their mothers to report heightened levels of maladaptive ER strategies. These results are in line with previous findings of a relation between maladaptive ER and psychopathological symptoms (e.g. [25, 42, 43]). Maladaptive ER concerning anxiety impairs coping with anxiety and increases avoidance in the face of anxiety-provoking situations. Thus, if mothers display maladaptive ER, it is highly likely that they shy away from anxiety-provoking situations both for themselves and for their child (e.g. [12, 44]) by avoiding the situation or overprotecting their child.

As indicated by previous research [39], age significantly influenced children’s adaptive ER. This may be explained by a closer inspection of the strategies we examined. In the present study, ER strategies consisted largely of cognitive strategies. In contrast to behavioral strategies, cognitive strategies such as self-degradation, acceptance and reappraisal demand an advanced ability to reflect upon cognitions and conceptualizations [45]. ER is organized by top-down, cortical brain networks that are presumed to mature into the early 20s (e.g. [46]). Thus, while still in the process of ER development, children may start to gradually use cognitive strategies, with early youth being the crucial stage of adaptation to internal and higher cognitive ER strategies.

Of note, maternal maladaptive ER did not explicitly predict child maladaptive ER but showed trend effects when regarded in relation to age. This seemingly disagrees with Bariola, Hughes, et al. [20], who report maternal suppression to predict child suppression. Furthermore, in contrast to this study [20] we found an influence of children’s age on the prediction of ER, however only for adaptive child ER. One reason for the discrepant results thus may be that the present study included a greater number of ER strategies than Bariola et al. [20]. In addition, the children’s mean age in the Bariola et al. study (15 years) was substantially higher than the mean age in our sample (11 years). Overall, we found child adaptive ER to be positively predicted by age, but this relation interacted in an unpredicted manner with maternal maladaptive ER strategies. In older children higher maternal maladaptive ER is related to lower child adaptive ER, as might be explained by the child’s development in an environment that does not enable adaptive ER strategy development. But for younger children, this effect is inversed, such that more maternal maladaptive ER strategies are related to more adaptive child ER strategies. In the course of cognitive and emotional development, use of ER strategies changes from external to internal ER (e.g. [12, 45]). Thus, a stronger maternal influence in older children and their adjustment to a parental role model seems plausible. Later on, if both mothers and children use more maladaptive ER strategies when suffering from anxiety symptoms, the relation increases in importance. In other words, mothers using maladaptive ER strategies may provide an environment which complicates the development of adaptive ER strategies in children. However, use of ER strategies in general increases with age, especially considering the use of adaptive strategies. Nevertheless, given our range of age at a very specific phase of childhood (middle to late childhood, excluding adolescence), these findings require further examination as there was no a priori assumption concerning age; thus more research specifically concerning influences of age is necessary.

In relation to the tripartite model of familial influence [15], the current results point to parental as well as child characteristics to be important towards the child’s ER. While age as a child characteristic modulated the relation between maternal ER and child ER, maternal social anxiety seems to be relevant for maternal ER which then shapes the emotional climate of the family.

From a clinical perspective, it is important to note that children with SAD did not lack adaptive ER strategies facing anxiety. Therefore, specific interventions could aim to decrease maladaptive ER strategies by diverting the focus from maladaptive to adaptive strategies. Such specific ER training could be implemented as an additional component in standard cognitive behavioral therapy, which has previously been shown to be effective with regard to reducing SAD symptoms [47]. Research on inclusion of parents in child anxiety treatment has to date been inconclusive. Some studies show no superiority of family-based treatment (e.g. [48, 49]), while others have found slightly more positive effects (e.g. [50, 51]). As parents form a central part of the child’s environment during and after treatment, inclusion of parents might help to stabilize and further increase therapeutic effects [52]. Methods of parental participation in therapy have varied, including family sessions (e.g. [50]), parents as observers during child therapy (e.g. [49]), and parent-only sessions (e.g. [51]). Thus, even though overall meta-analyses do not show an additional effect of including parents in child anxiety therapy, research on specific aspects of children with anxiety disorders and their families is necessary.

Our results indicate that parental involvement in therapy could be useful if targeted more specifically. This could include a specific assessment of maternal adaptive and maladaptive ER, with the aim to specifically increase usage of adaptive ER, thereby possibly reducing child social anxiety symptoms. Further, maternal social anxiety itself could be addressed to decrease its negative influence on child ER deficits. Importantly, further studies addressing the role of model learning and emotion-directed coaching [15, 53, 54] are needed to better understand the role of parental influence on child psychopathology and ER.

Overall, the current study points to ER as central in anxiety for both children and mothers. Still, accumulation of both anxiety and ER symptoms show the importance to focus not only on the anxious child but also on environmental factors in the family. Further, results on age point to the relevance of development in emotion regulation, especially in social anxiety. However, no causal inferences about the mother-child relationship can be drawn as we rely on cross-sectional data. Therefore, only future studies including longitudinal data could shed light on important steps in child development of anxiety and ER. Additionally, larger samples including a wider range of age would clarify the current results of age as a significant factor by increasing power (e.g. [41]). A larger sample including youth would further allow analyzing of possible relations between maternal emotion dysregulation and child social anxiety mediated by child emotion dysregulation which was limited by sample size in the current study. Further, ER is typically assessed by questionnaires which include possibilities of reporting biases (e.g. [55]). Different assessments such as experimental paradigms measuring more objective implicit ER could thus be of interest (e.g. [56, 57]). Maternal questionnaires could not be based on validated assessments of ER as no parallel assessment of child and adult ER currently exists. However, the chosen method of assessment allows to compare maternal and child ER directly as the same, manifold strategies were assessed in both mother and child. Still precautions have to be heeded as, although this adapted instrument correlated with the ERQ (with a weaker correlation for the subscale suppression), there are doubts if the newly constructed instrument has proper construct-validity. Against this, internal consistency in the sample was high; however, future studies should validate this measure against other explicit measures of ER (e.g., the Cognitive Emotion Regulation Questionnaire [58]) or implicit measures of ER (e.g. neural correlates [57]). Furthermore, our sample consisted of children with diagnosed social anxiety disorder which allows inferences to clinical levels of anxiety. The current study focuses on social anxiety as the aim was to achieve specific conclusions starting from a narrow perspective. In a next step, a transfer to other anxiety disorders or general psychopathology would prove further interesting insights.

In conclusion, our data reveal that both child and maternal ER is a central component of child SAD. However, age and, thus, development seems to play a crucial role in familial relations of emotion dysregulation possibly pointing to changes through development and an adaptation to maternal emotion regulation patterns over time. Familial accumulation of social anxiety symptoms and emotion dysregulation has often been assumed theoretically. The current results allow for one of the first empirical findings of maladaptive and adaptive ER in child SAD and extend findings to a more developmental perspective by including age.
